# Supplementary material for: Development and evaluation of the automated multipurpose molecular testing system PCRpack for high-throughput SARS-CoV-2 testing
Source: Microbiol Spectr. 2023 Nov 9;11(6):e02716-23. doi: 10.1128/spectrum.02716-23 (PMC10715159; doi:10.1128/spectrum.02716-23)
Supplement: Tables S1, Figures S1 to S4 — Supplemental Tables and Figures. [file spectrum.02716-23-s0001.docx]

**Development and evaluation of the automated multipurpose molecular testing system PCRpack for high-throughput SARS-CoV-2 testing**

**Supplementary material**

**Table S1. Analytical sensitivity of the PCRpack system.**

| Specimen type | Limit of detection, genome copies/mL sample | Viral genome copies/mL sample, positive rate (no. of replicates, positive/tested) | | |
| --- | --- | --- | --- | --- |
|  |  | 2,500 | 1,000 | 500 |
| Saliva swab | 1,000 | 100% (20/20) | 100% (20/20) | 75% (15/20) |
| Nasal swab | 1,000 | 100% (20/20) | 95% (19/20) | 60% (12/20) |

**Figure S1. Workflow of RT-PCR testing by the manual method.** The workflow was divided into 6 procedures, and each procedure included several test steps. Broken line between steps indicates that the below and above steps were performed simultaneously. Tube barcodes were read one by one using a barcode reader and SimpPCR.

**
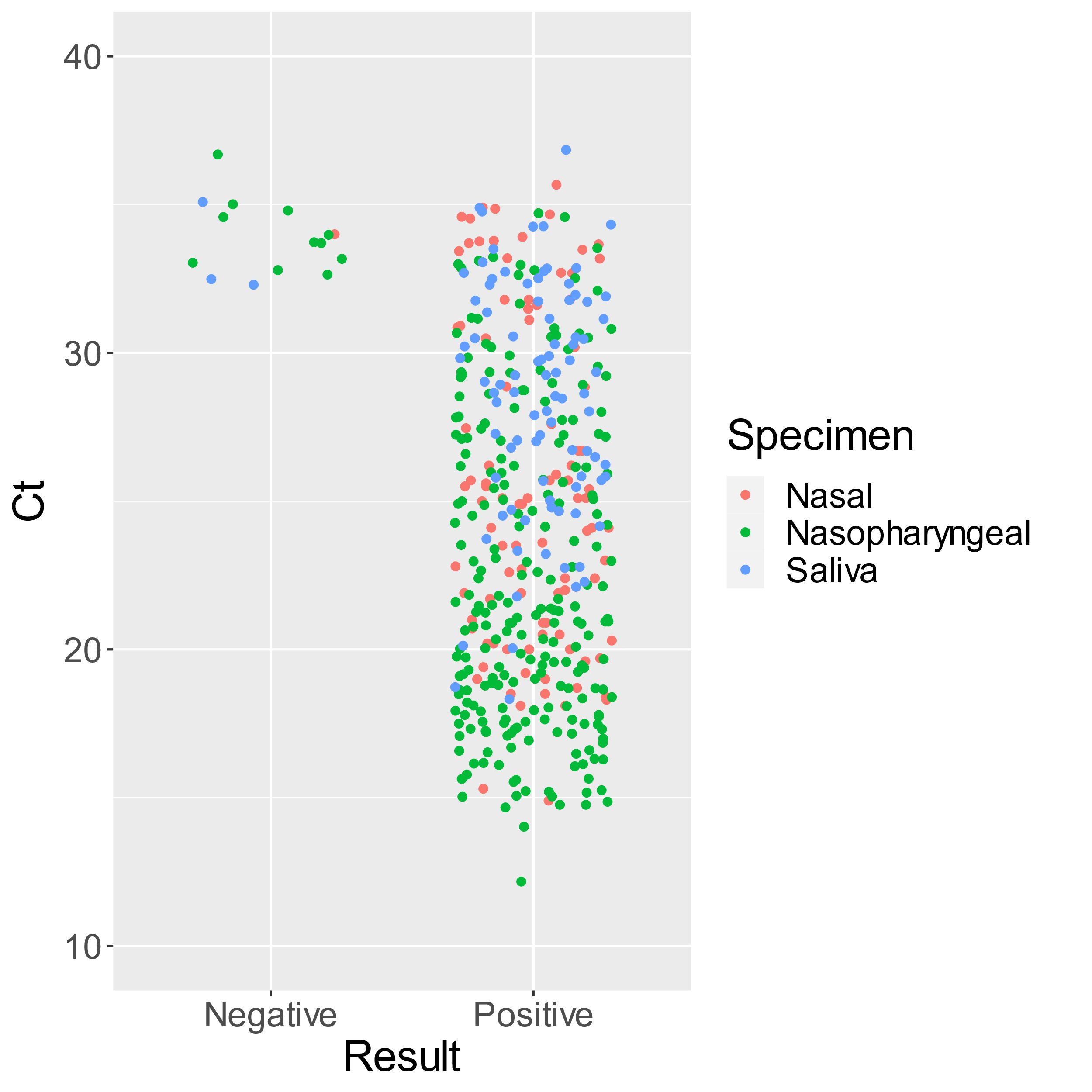
**

**Figure S2. Ct values of the 436 reference assay-positive samples according to the results of PCRpack.** The mean Ct value was 24.7 (range, 12.2–36.8). The Ct values of 15 samples that were negative by PCRpack were high (mean, 33.9; range, 32.3–36.7). Raw data are available in Dataset S1.

**Figure S3. Hands-on and hands-off timelines of multiple test batches assessed by PCRpack and the manual method within an 8-hour~~-day~~ shift of one operator.** PCRpack was estimated to test a maximum of 564 samples (6 batches) in a total of 7 hours and 50 minutes (2 hours and 34 minutes hands-on time). The manual method was estimated to test a maximum of 376 samples (4 batches) in a total of 7 hours and 15 minutes (5 hours and 36 minutes hands-on time).

**Figure S4. Hands-on and hands-off timelines of multiple test batches assessed by PCRpack with an additional PCR instrument within an 8-hour shift of one operator.** The PCRpack system configured with 2 PCR instruments was estimated to test a maximum of 752 samples (8 batches) in a total of 7 hours and 46 minutes (3 hours and 25 minutes hands-on time).
